# Knowledge, attitudes, and practice regarding rehabilitation among postoperative patients with spinal degenerative diseases: a cross-sectional study

**DOI:** 10.3389/fpubh.2026.1885115

**Published:** 2026-09-03

**Authors:** Mingming Liu, Xiaoye Guo, Yutong Bi, Doudou Chen, Qiao Chen, Jinqing Zhang, Ying Han, Bing Han

**Affiliations:** 1 Department of Spine Surgery, Beijing Jishuitan Hospital, Capital Medical University, Beijing, China; 2 Department of Spine Surgery, Jinan Third People's Hospital, Jinan, China; 3 School of Nursing, Capital Medical University, Beijing, China; 4 Department of Orthopedic, Miyun Hospital, Peking University First Hospital, Beijing, China; 5 Department of Orthopaedic Surgery, Beijing Jishuitan Hospital, Capital Medical University, Beijing, China; 6 Department of Nursing, Beijing Jishuitan Hospital, Capital Medical University, Beijing, China

**Keywords:** knowledge, attitudes, practice, patient compliance, postoperative care, rehabilitation, spinal diseases, surveys and questionnaires

## Abstract

**Background:**

This study aimed to investigate postoperative patients’ knowledge, attitudes, and practice (KAP) regarding spinal degenerative diseases and rehabilitation training.

**Methods:**

This single-center cross-sectional study was conducted from July 1, 2025, to September 30, 2025, at Beijing Jishuitan Hospital, Capital Medical University. The questionnaire was developed through two rounds of Delphi consultation with seven experts, pilot-tested in 35 patients, and evaluated using confirmatory factor analysis (CFA).

**Results:**

Among 382 included patients (median age, 59 years [P25, P75: 49, 66]; 54.5% male), the median (P25, P75) knowledge, attitude, and practice scores were 21 (14, 28) (possible range: 0–39), 43 (40, 49) (10–50), and 33 (26, 40) (10–50), respectively. CFA supported the three-factor structure (CMIN/DF = 2.607, IFI = 0.915, TLI = 0.905, CFI = 0.915, and RMSEA = 0.065). SEM showed positive path coefficients from knowledge to attitude (*β* = 0.707, *p* = 0.011), knowledge to practice (*β* = 0.493, *p* = 0.014), and attitude to practice (*β* = 0.269, *p* = 0.008). A positive indirect association between knowledge and practice through attitude was also observed (*β* = 0.190, *p* = 0.006).

**Conclusion:**

Participants demonstrated moderate knowledge, positive attitudes, and moderate practice behaviors. The findings identify an attitude–practice gap and support evaluating structured follow-up and practical barrier-focused strategies to help patients sustain rehabilitation behaviors.

## Background

Spinal degenerative diseases, including lumbar disc herniation, cervical spondylosis, and lumbar spinal stenosis, impose a substantial global health burden and remain leading causes of chronic pain and disability among adults (1, 2). Epidemiological studies demonstrate that these conditions are highly prevalent in aging societies. In a large U.S. Medicare cohort, 27.3% of older adults were diagnosed with degenerative disc disease, with prevalence increasing markedly with age (3). In China, low back pain frequently linked to lumbar degenerative pathology affected nearly 100 million individuals in 2021 and continues to rise with population aging (4). Lumbar disc herniation is particularly common, accounting for approximately 77.8% of lumbar degenerative presentations in clinical cohorts (5). These disorders often lead to persistent pain, limited mobility, sensory deficits, and a profound decline in quality of life (6). While surgical strategies such as decompression or fusion are widely used for refractory symptoms, postoperative recovery varies, and structured rehabilitation remains critical for restoring spinal stability, preventing complications, and optimizing long-term outcomes (7).

The knowledge, attitudes, and practice (KAP) framework provides a structured approach for assessing how patients understand their disease, perceive rehabilitation, and engage in health-promoting behaviors (8). The KAP model posits that adequate disease-related knowledge can shape patients’ health beliefs, which subsequently influence their behavioral adherence, making it a relevant tool for evaluating postoperative self-management (9). Recent KAP-based investigations in musculoskeletal and orthopedic conditions indicate that many patients exhibit limited knowledge of disease mechanisms, rehabilitation principles, and long-term self-management, even though they report generally positive attitudes toward recovery (5, 10). Among individuals with lumbar degenerative disorders, inadequate health literacy and low adherence to prescribed rehabilitation exercises have been identified as key obstacles to optimal postoperative outcomes (11). Comparable knowledge–practice gaps have also been documented in patients undergoing surgeries for conditions such as rotator cuff tears or osteoporosis-related complications, where supportive attitudes often do not translate into sustained rehabilitation behaviors (10, 12). Despite growing interest in KAP research within orthopedic settings, evidence specifically addressing postoperative patients with spinal degenerative diseases remains limited. This gap in knowledge restricts the development of targeted educational strategies and limits understanding of the behavioral and cognitive barriers that influence postoperative rehabilitation participation.

Therefore, this study aims to investigate postoperative patients’ knowledge, attitudes, and practice regarding spinal degenerative diseases and rehabilitation training, and to identify factors associated with these KAP dimensions. By characterizing disease-related understanding and rehabilitation behaviors in this population, the study seeks to provide evidence to inform tailored educational and behavioral interventions. Strengthening KAP levels among postoperative patients may ultimately improve rehabilitation adherence, enhance functional recovery, and support more effective long-term management of spinal degenerative diseases.

## Methods

### Study design and participants

This single-center cross-sectional study was conducted from July 1, 2025, to September 30, 2025, at Beijing Jishuitan Hospital, Capital Medical University, and enrolled postoperative patients diagnosed with spinal degenerative diseases. Inclusion criteria were: (1) patients attending postoperative outpatient follow-up approximately 3 months after surgery for a degenerative spinal disease; (2) age 18–75 years; and (3) provision of informed consent and voluntary participation. Patients were excluded if, as assessed by a trained investigator, they were unable to understand the study requirements, complete the questionnaire, or follow standardized instructions because of clinically evident cognitive impairment (e.g., Alzheimer’s disease or vascular dementia), language or communication barriers, confusion, or severe psychiatric symptoms (e.g., a severe anxiety or depressive episode). Physical difficulty with writing alone did not result in exclusion because standardized assistance was available; exclusion applied only when valid questionnaire completion remained impossible despite such assistance. The study protocol was approved by the Ethics Committee of Beijing Jishuitan Hospital, Capital Medical University (approval reference: Jishuitan Ethics Review [K2024] No. [001]-01), and all participants provided written informed consent before participation. Personally identifiable information was replaced with unique study codes, data were stored in an encrypted, access-controlled database available only to authorized core research personnel, and only aggregated results were reported.

### Sample size

Sample size was estimated using the single-proportion formula *n* = (Z_(1-α/2)/δ)^2 × *p* × (1-*p*), where n is the required total sample size, α is the type I error rate, δ is the allowable error, and p is the anticipated proportion. With α = 0.05 (Z_(1-α/2) = 1.96), δ = 0.05, and *p* = 0.50 to provide the maximum variance, the estimated target was *n* = (1.96/0.05)^2 × 0.50 × (1–0.50) = 384.16, or approximately 384 participants. A total of 382 valid questionnaires were included (99.5% of the nominal target), corresponding to an effective two-sided margin of error of approximately 5.01% under the same conservative assumptions.

### Questionnaire development, validation, and survey procedures

A self-developed questionnaire was employed for data collection. Its initial content was informed by relevant guidelines and literature (13, 14). Content validity was evaluated by refining the questionnaire through two rounds of Delphi consultation with seven experts: one chief and one associate chief spinal surgeon, one associate chief rehabilitation physician, one senior rehabilitation therapist, one associate chief spinal-surgery nurse, and two senior spinal-surgery nurses. The experts evaluated item relevance, accuracy, completeness, clarity, and comprehensibility through written consultation and e-mail feedback. Their recommendations led to the addition of potentially relevant background variables, such as disease duration and education level, and revisions to the wording and response format of the practice items. A pilot study in 35 patients, who were not included in the main study sample, yielded a Cronbach’s alpha of 0.947, indicating excellent internal consistency. Construct validity was evaluated in the final analytic sample: the Kaiser–Meyer–Olkin value was 0.947 and Bartlett’s test of sphericity was significant (*p* < 0.001). CFA supported the prespecified three-factor structure; standardized factor loadings ranged from 0.405 to 0.863.

The final Chinese-language instrument comprised four sections: 25 items on demographic and clinical characteristics, 13 knowledge items, 10 attitude items, and 10 practice items. Knowledge responses were scored on a four-point scale: 3 for ‘well informed,’ 2 for ‘moderately informed,’ 1 for ‘have heard of it,’ and 0 for ‘unfamiliar,’ yielding a possible range of 0–39. Attitude and practice items were rated on five-point Likert scales, yielding possible scores of 10–50 for each dimension. Scores were categorized as follows: knowledge, insufficient (0–13), moderate (14–26), or adequate (27–39); attitude, negative (10–25), neutral (26–35), or positive (36–50); and practice, inadequate (10–25), moderate (26–35), or proactive (36–50) (15, 16).

Participants were recruited by convenience sampling during the approximately 3-month postoperative outpatient follow-up. A trained physician and two trained nurses administered either a paper questionnaire or an electronic questionnaire accessed by QR code. Participants completed the questionnaire individually in a private room while waiting for the consultation. When an item was not understood, a trained investigator provided a standardized explanation or assisted with completion without suggesting a response. Quality-control measures included restricting duplicate electronic submissions, requiring responses to all items, applying a minimum completion time, checking logical consistency, and excluding incomplete or clearly contradictory questionnaires. Questionnaires completed in less than 180 s, containing logically inconsistent responses, or showing the same response to all items in any KAP section were considered invalid.

### Statistical analysis

Statistical analyses were performed using SPSS version 26.0 and IBM SPSS Amos version 26.0 (IBM Corp., Armonk, NY, USA). Normality of continuous variables was assessed using the one-sample Kolmogorov–Smirnov test. Normally distributed continuous variables are presented as mean ± SD, whereas nonnormally distributed variables are presented as median (P25, P75); categorical variables are presented as frequencies and percentages. Because the knowledge, attitude, and practice scores were nonnormally distributed, between-group comparisons used the Mann–Whitney U test for two groups and the Kruskal–Wallis test for more than two groups. Categorical variables were compared using the chi-square test or Fisher’s exact test, as appropriate. Sampling adequacy was evaluated using the Kaiser–Meyer–Olkin test and Bartlett’s test of sphericity. CFA was used to evaluate the three-factor measurement structure. SEM with bootstrap estimation was used to examine hypothesized associations and indirect pathways among knowledge, attitudes, and practice. Because the data were cross-sectional, path coefficients were interpreted as associations rather than causal effects. Model fit was assessed using CMIN/DF, RMSEA, IFI, TLI, and CFI; CMIN/DF < 3, RMSEA<0.08, and IFI, TLI, and CFI values around or above 0.90 were considered indicative of acceptable fit. All statistical tests were two-sided, and *p* < 0.05 was considered statistically significant.

## Results

Initially, this study collected a total of 404 questionnaires. The following data were excluded: (1) one case with abnormal height and weight; (2) four cases with all items in the Knowledge section marked as “not clear” and one case with all items marked as “have heard of it”; (3) one case with all items in the Attitude section marked as “neutral”; (4) 10 cases with all items in both the Attitude and Practice sections marked as “strongly agree”; (5) five cases with all items in the Practice section marked as “neutral.” Ultimately, 382 valid questionnaires remained, with an effective rate of 94.55%.

### Basic information on the population and KAP score

This study included 382 postoperative patients with spinal degenerative diseases, with a median age of 59 years (P25, P75: 49, 66) and a slight male predominance (54.5%). The most common diagnoses were lumbar disc herniation (25.7%) and lumbar spinal stenosis (23.6%). Most patients had an education level of middle school or below (57.9%) and a monthly income below 6,000 RMB (67.0%). Thoracic or lumbar internal fixation/fusion was the most frequent surgical procedure (52.9%), and the majority had a disease duration of ≤5 years (74.1%). The one-sample Kolmogorov–Smirnov test indicated that the knowledge, attitude, and practice scores were nonnormally distributed (all *p* < 0.001). Their median (P25, P75) scores were 21 (14, 28), 43 (40, 49), and 33 (26, 40), respectively. Knowledge scores differed significantly by education level (*p* < 0.001), monthly income (*p* < 0.001), drinking habits (*p* = 0.036), engagement in heavy physical labor (*p* = 0.037), prolonged heavy physical labor (*p* = 0.006), prolonged desk work (*p* < 0.001), and history of spine-related surgery (*p* < 0.001). Attitude scores varied significantly with education level (*p* < 0.001), monthly income (*p* < 0.001), engagement in prolonged heavy physical labor (*p* < 0.001), prolonged desk work (*p* < 0.001), and history of spine-related surgery (*p* = 0.016). Practice scores showed significant associations with gender (*p* = 0.018), education level (*p* < 0.001), monthly income (*p* < 0.001), marital status (*p* = 0.032), engagement in prolonged heavy physical labor (*p* = 0.013), prolonged desk work (*p* = 0.003), and history of spine-related surgery (*p* = 0.034) (Table 1).

**Table 1:** Demographic characteristics and KAP scores.

Variables	*n* (%) or median (P25, P75)	Knowledge, median (P25, P75)	*p*	Attitude, median (P25, P75)	*p*	Practice, median (P25, P75)	*p*
Total score		21 (14, 28)		43 (40, 49)		33 (26, 40)	
Age, years (range: 18–75)	59 (49, 66)						
Gender			0.101		0.061		0.018
Male	208 (54.45%)	23 (14, 28)		43 (40, 49)		35 (27, 40)	
Female	174 (45.55%)	19 (14, 26)		42 (40, 48)		32 (25, 39)	
Diagnosis			0.12		0.117		0.227
Cervical disc herniation	47 (12.30%)	24 (16, 30)		42 (40, 50)		35 (31, 40)	
Cervical spinal stenosis	68 (17.80%)	24 (16, 28.75)		45 (40, 49.75)		32 (25.25, 39)	
Lumbar spondylolisthesis	57 (14.92%)	21 (16, 26)		43 (40, 48.5)		34 (28, 40)	
Lumbar disc herniation	98 (25.65%)	22 (14, 29.25)		43 (39.75, 49)		34 (26.75, 41.25)	
Lumbar spinal stenosis	90 (23.56%)	18 (12, 26)		41 (39, 46)		29.5 (25, 38)	
Other	8 (2.09%)	18 (10.75, 27.5)		41 (40, 44.25)		40.5 (23.25, 45)	
Thoracic spinal stenosis	14 (3.66%)	17 (12, 28.75)		40.5 (40, 50)		33 (25.75, 40)	
Duration			0.496		0.756		0.436
≤5 years	283 (74.08%)	20 (14, 28)		43 (40, 49)		32 (25, 40)	
>5 years and ≤10 years	48 (12.57%)	20.5 (15.25, 26.75)		43 (40, 50)		35 (28, 40.75)	
>10 years	51 (13.35%)	24 (14, 30)		44 (40, 49)		34 (27, 39)	
Surgical procedure			0.156		0.173		0.088
Anterior cervical internal fixation/fusion	68 (17.80%)	23.5 (15, 28)		43 (40, 49)		34.5 (30, 40)	
Thoracic or lumbar internal fixation/fusion	202 (52.88%)	20 (14, 26)		42.5 (40, 49)		33 (26.75, 40)	
Minimally invasive lumbar discectomy (e.g., transforaminal endoscopic discectomy/microendoscopic discectomy)	52 (13.61%)	22 (11.25, 28)		41 (39, 48.5)		29.5 (24, 38)	
Other spinal surgeries	60 (15.71%)	24 (16.25, 29.75)		45 (40, 49.75)		31.5 (25, 39.75)	
BMI			0.655		0.247		0.654
18.5–23.9	117 (30.63%)	20 (15, 28.5)		43 (40, 49)		33 (26, 40.5)	
24 ≤ BMI < 28	172 (45.03%)	21 (13, 26)		42 (40, 48)		34 (24.25, 39.75)	
BMI ≥ 28	93 (24.35%)	23 (15, 28)		44 (40, 49)		34 (27, 40)	
Education			<0.001		<0.001		<0.001
Middle school or below	221 (57.85%)	17 (13, 24.5)		41 (39, 46)		30 (25, 37)	
Bachelor’s degree or above	161 (42.15%)	26 (20.5, 31.5)		48 (40.5, 50)		38 (30, 42.5)	
Monthly income per capita			<0.001		<0.001		<0.001
<3,000	120 (31.41%)	17 (13, 26)		41 (40, 46.75)		32 (25, 38)	
3,000–6,000	136 (35.60%)	18.5 (13, 26)		41 (39, 48)		31 (25, 38.75)	
6,000–10,000	87 (22.77%)	24 (18, 31)		45 (40, 50)		36 (28, 42)	
>10,000	39 (10.21%)	27 (25, 32)		49 (43, 50)		39 (33, 44)	
Marital status			0.198		0.668		0.032
Married	356 (93.19%)	21 (14.25, 28)		43 (40, 49)		33 (27, 40)	
Other	26 (6.81%)	16.5 (12.75, 26)		42 (39, 48.5)		27 (22.75, 37.5)	
Smoking			0.465		0.073		0.117
Never	230 (60.21%)	20 (14, 28)		42.5 (40, 48.25)		32 (26, 40)	
Quit	50 (13.09%)	25 (14,28)		47 (40,49)		38 (26.75,41)	
Current smoker	87 (22.77%)	19 (14,27)		42 (40,49)		32 (25,38)	
Occasional smoker	15 (3.93%)	26 (16,28)		46 (41,50)		38 (33,40)	
Drinking alcohol			0.036		0.103		0.263
Never	208 (54.45%)	19 (13, 27)		41 (39, 48)		31 (26, 40)	
Quit	26 (6.81%)	26 (17.75, 28)		47 (40.75, 49.25)		38 (26.75, 40)	
Current drinker	47 (12.30%)	20 (13, 26)		43 (40, 48)		33 (25, 40)	
Occasional drinker	101 (26.44%)	24 (16, 28)		44 (40, 49)		35 (28, 40)	
Engage in heavy physical labor			0.037		0.071		0.107
Yes	41 (10.73%)	17 (11, 25.5)		41 (39, 47)		30 (23.5, 39.5)	
No	341 (89.27%)	21 (14.5, 28)		43 (40, 49)		33 (26, 40)	
Engage in prolonged heavy physical labor			0.006		<0.001		0.013
Yes	139 (36.39%)	19 (13, 26)		41 (39, 48)		31 (25, 39)	
No	243 (63.61%)	24 (15, 28)		44 (40, 49)		35 (27, 40)	
Engage in prolonged desk work			<0.001		<0.001		0.003
Yes	101 (26.44%)	26 (16, 31.5)		47 (40, 50)		36 (29, 41.5)	
No	281 (73.56%)	19 (14, 26)		42 (40, 48)		32 (25, 39)	
Long-term and physically exhausting with frequent jolting work			0.085		0.553		0.172
Yes	45 (11.78%)	24 (16, 31)		43 (40, 49.5)		35 (27.5, 41.5)	
No	337 (88.22%)	20 (14, 27)		43 (40, 49)		33 (26, 40)	
Acute trauma involving the spine			0.071		0.114		0.228
Yes	49 (12.83%)	23 (17.5, 31.5)		45 (40.5, 49)		35 (28.5, 42.5)	
No	333 (87.17%)	21 (14, 27)		42 (40, 49)		33 (26, 40)	
Other spine-related diseases							
Lumbar disc herniation			0.537		0.945		0.318
Yes	56 (14.66%)	20 (14, 28)		42.5 (40, 49)		33 (26, 39.25)	
No	326 (85.34%)	23 (14, 27.75)		43.5 (40, 48)		35.5 (28, 40)	
Lumbar spinal stenosis			0.558		0.48		0.685
Yes	22 (5.76%)	20 (14, 28)		43 (40, 49)		33 (26, 40)	
No	360 (94.24%)	24 (16.75, 26.5)		43.5 (40, 49)		33 (24, 39.25)	
Spine-related surgery			<0.001		0.016		0.034
Yes	38 (9.95%)	28 (22, 33)		47 (40.75, 50)		37 (29, 41.25)	
No	344 (90.05%)	20 (14, 27)		42 (40, 48.75)		32.5 (26, 40)	

### Distribution of responses to knowledge, attitude, and practice

The distribution of knowledge dimensions showed that the three questions with the lowest number of participants choosing the ‘Very familiar’ option were ‘Targeted muscle training can relieve pain and other discomfort.’ (K9) with 7.59%, ‘The goal of rehabilitation training is to strengthen the core spinal muscles, improve spinal stability, protect the spine, prevent muscle fatigue and atrophy, and reduce discomfort such as neck/shoulder pain and low back and leg pain.’ (K7) with 10.73%, and ‘After surgery for degenerative spinal diseases, complications may occur, such as wound infection, nerve injury (worsening numbness or pain in the limbs), cerebrospinal fluid leakage, wound hematoma, or respiratory difficulty (cervical spine).’ (K3) with 11.52% (Table 2).

**Table 2:** Distribution of knowledge dimension responses.

Items	*n* (%)			
	Very familiar	Generally familiar	Have only heard of it	Not familiar
1. Degenerative spinal diseases refer to conditions in which long-term pressure and wear on the spine lead to structural and functional changes.	72 (18.85%)	166 (43.46%)	89 (23.30%)	55 (14.40%)
2. Degenerative spinal diseases may be related to aging, poor posture, heavy physical labor, and other factors.	86 (22.51%)	163 (42.67%)	97 (25.39%)	36 (9.42%)
3. After surgery for degenerative spinal diseases, complications may occur, such as wound infection, nerve injury (worsening numbness or pain in the limbs), cerebrospinal fluid leakage, wound hematoma, or respiratory difficulty (cervical spine).	44 (11.52%)	164 (42.93%)	115 (30.10%)	59 (15.45%)
4. Neck and shoulder pain or low back pain are common symptoms. Many patients first experience recurrent neck/shoulder or low back pain, followed by pain in both upper limbs, all limbs, or both lower limbs. Radicular pain is very common.	66 (17.28%)	157 (41.10%)	106 (27.75%)	53 (13.87%)
5. Symptoms such as numbness in the upper limbs, difficulty using chopsticks or writing smoothly, difficulty fastening shirt buttons, or inability to lift the arm may be related to cervical spine disease.	74 (19.37%)	119 (31.15%)	107 (28.01%)	82 (21.47%)
6. Symptoms such as weakness in the toes, numbness in the toes, difficulty lifting the legs, or stumbling while walking may be related to lumbar spine disease.	115 (30.10%)	158 (41.36%)	69 (18.06%)	40 (10.47%)
7. The goal of rehabilitation training is to strengthen the core spinal muscles, improve spinal stability, protect the spine, prevent muscle fatigue and atrophy, and reduce discomfort such as neck/shoulder pain and low back and leg pain.	41 (10.73%)	161 (42.15%)	93 (24.35%)	87 (22.77%)
8. In the early postoperative period (rehabilitation may begin on the day of surgery), moderate activities such as bed turning and limb movements help prevent deep venous thrombosis of the lower limbs, promote gastrointestinal motility, and prevent abdominal distension.	77 (20.16%)	183 (47.91%)	98 (25.65%)	24 (6.28%)
9. Targeted muscle training can relieve pain and other discomfort (e.g., cervical muscle training can relieve postoperative neck and shoulder pain after cervical surgery; lumbar and back muscle training can improve postoperative low back pain after lumbar surgery).	29 (7.59%)	169 (44.24%)	88 (23.04%)	96 (25.13%)
10. After surgery for degenerative spinal diseases, increasing dietary intake of protein, calcium, and other nutrients can improve the nutrition of spinal muscles and enhance muscle quality.	126 (32.98%)	183 (47.91%)	48 (12.57%)	25 (6.54%)
11. Rehabilitation training requires long-term adherence to achieve good results; short-term or irregular exercise is ineffective.	67 (17.54%)	169 (44.24%)	85 (22.25%)	61 (15.97%)
12. Postoperative use of braces (e.g., cervical collar, lumbar brace) can support the spine and reduce pain, but bracing should not exceed 3 months, as prolonged use may lead to muscle atrophy, weakened strength, and increased risk of spinal instability, neck/shoulder pain, or low back pain.	70 (18.32%)	176 (46.07%)	89 (23.30%)	47 (12.30%)
13. If you visit the Department of Rehabilitation Medicine and receive targeted evaluation of muscle function by a rehabilitation physician, followed by personalized guidance, long-term adherence, regular follow-up, and periodic adjustment of the rehabilitation plan can significantly reduce postoperative discomfort such as neck/shoulder pain and low back and leg pain.	23 (6.02%)	146 (38.22%)	85 (22.25%)	128 (33.51%)

Responses to the attitude dimension showed that only 33.51% strongly agreed that they are willing to spend time and effort to learn more about postoperative rehabilitation (A3), only 38.74% are very confident that rehabilitation training can help accelerate their postoperative recovery (A5), and only 41.36% strongly agreed that following the rehabilitation plan prescribed by the doctor will lead to good recovery outcomes (A2) (Table 3).

**Table 3:** Distribution of attitude dimension responses.

Items	*n* (%)				
	Strongly agree	Agree	Neutral	Disagree	Strongly disagree
1. I believe that actively cooperating with rehabilitation training is very important for postoperative recovery from degenerative spinal diseases.	160 (41.88%)	155 (40.58%)	61 (15.97%)	5 (1.31%)	1 (0.26%)
2. I believe that following the rehabilitation plan prescribed by the doctor will lead to good recovery outcomes.	158 (41.36%)	166 (43.46%)	54 (14.14%)	4 (1.05%)	0
3. I am willing to spend time and effort to learn more about postoperative rehabilitation.	128 (33.51%)	166 (43.46%)	80 (20.94%)	8 (2.09%)	0
4. I believe that support from family and friends is important for me during rehabilitation training.	173 (45.29%)	181 (47.38%)	25 (6.54%)	3 (0.79%)	0
5. I am confident that rehabilitation training can help accelerate my postoperative recovery.	148 (38.74%)	172 (45.03%)	59 (15.45%)	3 (0.79%)	0
6. I believe that society and medical institutions should increase public education about rehabilitation for degenerative spinal diseases.	207 (54.19%)	165 (43.19%)	10 (2.62%)	0	0
7. I believe that degenerative spinal diseases are common health issues and there is no need to feel inferior because of them.	206 (53.93%)	159 (41.62%)	11 (2.88%)	4 (1.05%)	2 (0.52%)
8. I do not feel embarrassed or uncomfortable when others know that I have a degenerative spinal disease.	215 (56.28%)	146 (38.22%)	15 (3.93%)	6 (1.57%)	0
9. I prefer to take an active and proactive approach in managing my degenerative spinal disease rather than waiting passively.	192 (50.26%)	169 (44.24%)	18 (4.71%)	3 (0.79%)	0
10. If people around me misunderstand degenerative spinal diseases, I am willing to explain and share correct information with them.	174 (45.55%)	181 (47.38%)	26 (6.81%)	1 (0.26%)	0

Responses to the practice dimension showed that only 4.71% always actively participate in spine rehabilitation or health education sessions or activities organized by hospitals or community groups (P9), only 7.85% always keep detailed records of their daily rehabilitation activities (P3), only 9.16% always perform each rehabilitation exercise carefully and ensure that all movements are performed correctly (P2) (Table 4).

**Table 4:** Distribution of practice dimension responses.

Items	*n* (%)				
	Always	Often	Sometimes	Rarely	Never
1. After surgery, I consistently follow the doctor’s recommended schedule and frequency for rehabilitation training.	43 (11.26%)	143 (37.43%)	109 (28.53%)	76 (19.90%)	11 (2.88%)
2. I perform each rehabilitation exercise carefully and ensure that all movements are performed correctly.	35 (9.16%)	137 (35.86%)	109 (28.53%)	90 (23.56%)	11 (2.88%)
3. I keep detailed records of my daily rehabilitation activities, such as training duration and training experiences.	30 (7.85%)	106 (27.75%)	97 (25.39%)	101 (26.44%)	48 (12.57%)
4. When discomfort occurs during rehabilitation training, I immediately handle it according to the doctor’s instructions.	69 (18.06%)	133 (34.82%)	121 (31.68%)	51 (13.35%)	8 (2.09%)
5. I actively seek medical consultations in the Department of Rehabilitation and regularly return to the hospital for postoperative follow-up visits.	74 (19.37%)	116 (30.37%)	96 (25.13%)	71 (18.59%)	25 (6.54%)
6. I proactively consult doctors or rehabilitation therapists about any problems encountered during rehabilitation training.	71 (18.59%)	115 (30.10%)	103 (26.96%)	67 (17.54%)	26 (6.81%)
7. I adjust my diet according to the doctor’s recommendations to promote postoperative recovery.	111 (29.06%)	158 (41.36%)	72 (18.85%)	33 (8.64%)	8 (2.09%)
8. In my daily life, I pay attention to maintaining correct posture (e.g., avoiding crossing legs, avoiding sleeping in vehicles, avoiding prolonged smartphone use) to reduce spinal stress.	58 (15.18%)	132 (34.55%)	149 (39.01%)	41 (10.73%)	2 (0.52%)
9. I actively participate in spine rehabilitation or health education sessions or activities organized by hospitals or community groups.	18 (4.71%)	61 (15.97%)	74 (19.37%)	148 (38.74%)	81 (21.20%)
10. I encourage people around me who also have degenerative spinal diseases to actively participate in rehabilitation training.	73 (19.11%)	119 (31.15%)	95 (24.87%)	65 (17.02%)	30 (7.85%)

### Correlations between KAP

Further correlation analysis revealed positive correlations between knowledge scores and attitude scores (r = 0.641, *p* < 0.001), as well as between knowledge scores and practice scores (*r* = 0.619, *p* < 0.001). Additionally, attitude scores were positively correlated with practice scores (r = 0.546, *p* < 0.001) (Table 5).

**Table 5:** Correlation analysis.

Dimension	Knowledge	Attitude	Practice
Knowledge	1		
Attitude	0.641 (*p* < 0.001)	1	
Practice	0.619 (*p* < 0.001)	0.546 (*p* < 0.001)	1

#### Questionnaire validation and structural equation modeling

**Figure 1:**
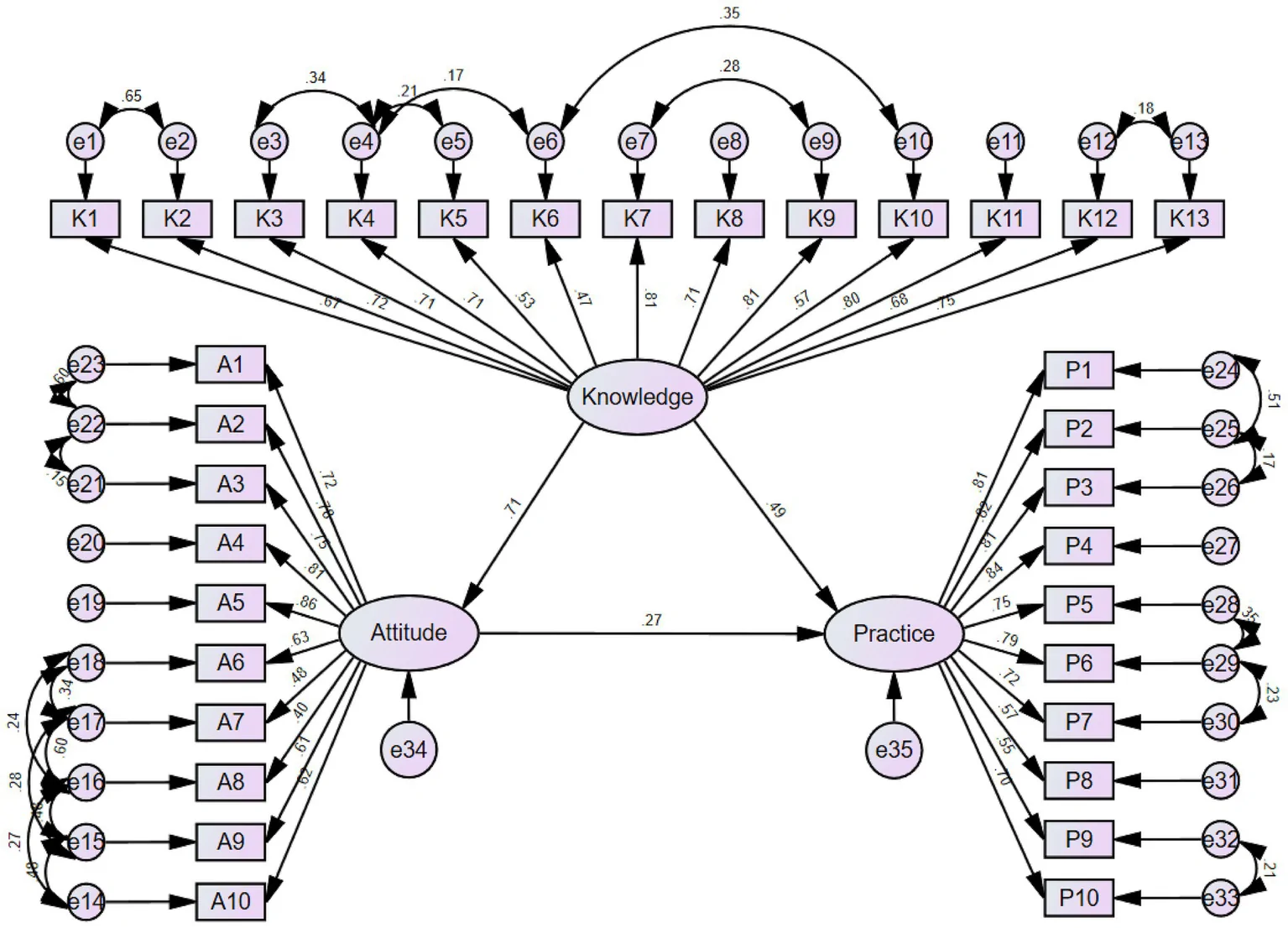
SEM model.

Questionnaire validation and SEM results showed that the Kaiser–Meyer–Olkin value was 0.947 and Bartlett’s test of sphericity was significant (*p* < 0.001). CFA demonstrated acceptable fit (CMIN/DF = 2.607, IFI = 0.915, TLI = 0.905, CFI = 0.915, and RMSEA = 0.065), supporting the three-factor structure (Supplementary Figure 1). The corrected SEM also showed acceptable fit (CMIN/DF = 2.564, IFI = 0.918, TLI = 0.907, CFI = 0.917, and RMSEA = 0.064; Supplementary Table S1). Positive path coefficients were observed from knowledge to attitude (*β* = 0.707, *p* = 0.011), knowledge to practice (*β* = 0.493, *p* = 0.014), and attitude to practice (*β* = 0.269, *p* = 0.008). A positive indirect association between knowledge and practice through attitude was also observed (*β* = 0.190, *p* = 0.006) (Table 6 and Figure 1). These paths describe statistical associations and do not establish temporal or causal relationships.

**Table 6:** Analysis of total, direct, and indirect associations.

Model paths	Standardized total association (95% CI)	*p*	Standardized direct path (95% CI)	*p*	Standardized indirect association (95% CI)	*p*
Knowledge → Attitude	0.707 (0.633–0.767)	0.011	0.707 (0.633–0.767)	0.011		
Knowledge → Practice	0.683 (0.579–0.743)	0.025	0.493 (0.352–0.636)	0.014	0.190 (0.093–0.286)	0.006
Attitude → Practice	0.269 (0.117–0.407)	0.008	0.269 (0.117–0.407)	0.008		

## Discussion

This study found that postoperative patients with spinal degenerative diseases demonstrated moderate knowledge and practice levels alongside positive attitudes. This reveals a significant attitude-practice gap, where favorable beliefs about rehabilitation do not fully translate into proactive behaviors. Implementing tiered, need-based rehabilitation education combined with structured follow-up systems may help convert positive attitudes into sustained, high-quality rehabilitation behaviors and support long-term functional recovery.

The co-occurrence of relatively good knowledge and attitudes with only moderate practice aligns with observations from other KAP-based rehabilitation studies in musculoskeletal populations, where patients often express willingness to recover but do not maintain regular therapeutic exercise or follow-up once formal supervision decreases (17–19). Related research suggests that this pattern frequently emerges when patients receive concentrated perioperative education without sufficient ongoing support for habit formation and problem-solving in daily life (17, 18). In studies of perioperative rehabilitation for joint disease, higher knowledge and positive expectations predicted greater initial engagement, but long-term adherence depended on the stability of routines and the practical feasibility of integrating exercises into everyday roles and responsibilities (19). The present findings appear to place postoperative spinal patients within this broader group of individuals who are cognitively prepared yet behaviorally under-supported, underscoring the need to view rehabilitation not only as a technical intervention but as a sustained behavior-change process that unfolds in domestic and occupational settings (17–19).

The knowledge profile revealed particular weaknesses in understanding of rehabilitation goals, targeted muscle training, and potential postoperative complications, despite generally adequate awareness of basic symptoms and risk factors. Studies in lumbar disc herniation and related spinal conditions have reported similar selective gaps, especially in domains that require patients to link abstract pathophysiology with concrete self-management tasks such as core muscle strengthening and staged activity progression (17, 20). Related research suggests that patients often remember simple diagnostic labels and short-term precautions but retain less information about long-term conditioning, neuromuscular mechanisms, and complication warning signs that are not immediately observable (20). Within this context, the relatively low familiarity with rehabilitation objectives and complication profiles in the present sample appears to reflect a wider educational pattern in which diagnostic explanation is prioritized over detailed discussion of long-term rehabilitation logic and risk management (17, 20).

Attitudes toward rehabilitation were generally positive, particularly regarding the importance of cooperation with training and the value of social and institutional support, yet the proportion of patients who expressed very strong willingness to invest time in learning or complete confidence in the benefits of training was more modest. Work in perioperative knee osteoarthritis rehabilitation has shown that even when most patients endorse rehabilitation in principle, only a subset report high self-efficacy and strong outcome expectations, and this subgroup tends to achieve superior functional gains (19). Qualitative and mixed-methods research on the postoperative course after lumbar fusion has highlighted that patients frequently encounter fluctuations in pain, fatigue, and psychological distress in the early months after surgery, which can erode initial optimism and create ambivalence about continued effort if perceived benefits are slow to emerge (21). Related research suggests that these shifts in mood and perceived controllability can weaken the translation from positive general attitudes to concrete action, particularly when follow-up encounters do not systematically address evolving doubts and emotional responses to setbacks (19, 21). The attitudinal profile observed here is therefore compatible with a scenario in which general endorsement of rehabilitation coexists with residual uncertainty about personal capacity and long-term benefit, creating a fragile motivational basis for sustained practice (19, 21).

The weakest dimension in this study was actual practice, especially behaviors requiring ongoing organization and initiative, such as active participation in health education sessions, detailed recording of rehabilitation activities, and careful execution of each exercise repetition. Related research suggests that such behaviors depend not only on motivation but also on time, memory, competing demands, and perceived value of specific self-monitoring tasks (22–24). Studies of compliance with postoperative physiotherapy across orthopedic populations have repeatedly documented substantial proportions of patients who either discontinue supervised therapy prematurely or inconsistently perform home exercises, with younger patients, those with higher role demands, and individuals experiencing early symptom relief being particularly prone to reduced adherence (22, 23). Classic work on exercise non-compliance in knee osteoarthritis similarly found that pain fluctuation, lack of immediate feedback, and competing responsibilities often outweighed patients’ stated intention to follow prescribed routines (24). Against this background, the low rates of meticulous record-keeping and proactive engagement with community-based rehabilitation in the present cohort are consistent with broader evidence that unsupervised, self-directed components of rehabilitation are especially vulnerable to attrition when structural supports are limited (22–24).

The observed correlations among knowledge, attitudes, and practice, together with the structural equation model, were consistent with a hypothesized KAP structure in which better knowledge was associated with more positive attitudes and more active rehabilitation behaviors. Because these data were collected cross-sectionally, the direction and temporality of the modeled pathways cannot be established. KAP-based interventions for lumbar disc herniation have shown that structured educational programs can improve knowledge and attitudes and, to a certain extent, enhance exercise adherence and functional outcomes, although behavioral change is rarely complete (17, 18). Perioperative KAP surveys in knee arthroplasty populations also report moderate associations between knowledge, self-efficacy, and reported exercise adherence, indicating that cognitive and motivational variables explain an important but partial proportion of behavioral variance (19). The present modeling results are consistent with this literature, suggesting that strengthening patient education and attitude formation may be helpful but is unlikely to be sufficient without additional environmental and organizational support.

Sociodemographic gradients in KAP scores, particularly by education level, income, and occupational characteristics, also align with wider evidence on health literacy and adherence. Studies in lumbar disc herniation and joint replacement populations describe higher knowledge and more favorable attitudes among patients with higher educational attainment and socio-economic resources, who typically have greater access to health information, more flexible work demands, and stronger confidence in interacting with professionals (17–19). Broader reviews of adherence across chronic conditions indicate that lower education, heavy physical labor, and unstable employment often coincide with reduced capacity to attend follow-up, limited time for structured exercise, and greater focus on immediate symptom relief rather than long-term risk reduction (23, 25). In addition, research on post-discharge spine surgery care suggests that patients discharged home with limited social or informational support may feel unprepared to manage pain and rehabilitation tasks, particularly when hospital stays are brief and instructions are dense or technical (26). The gradients seen in this survey therefore appear to reflect a combination of health literacy, work-related constraints, and differential access to supportive environments, rather than individual attitudinal deficits alone (23, 26).

The distribution patterns in the knowledge, attitude, and practice items also invite reflection on broader features of contemporary spine care. The low familiarity with the role of rehabilitation medicine services and targeted muscle training, alongside more confident endorsement of general statements about the importance of rehabilitation, resembles patterns described in observational studies of perioperative spine rehabilitation pathways, where patients report exposure to general advice but limited structured interaction with rehabilitation specialists before or shortly after surgery (26, 27). The present item-level findings, particularly the very low rates of active participation in health education activities and structured self-monitoring, appear to fit within this broader pattern of underdeveloped long-term rehabilitation infrastructure and variable continuity of care between hospital and community settings (26–28).

Taken together, these findings point toward the need for system-level adjustments that strengthen the organizational scaffolding around postoperative rehabilitation for spinal degenerative disease. Reviews of rehabilitation after lumbar spine surgery emphasize that programs tailored to surgical technique, preoperative function, and psychosocial context tend to produce better functional trajectories than generic exercise instruction, yet implementation remains uneven across institutions (27, 28). A recent synthesis of post-discharge interventions for degenerative spine surgery indicates that telemonitoring, structured telephone follow-up, and digital platforms can improve pain, function, and self-management for many patients, particularly when they provide timely feedback and clarify expectations about exercise progression and daily activity (26). Integrating such structured follow-up mechanisms into routine care, with explicit attention to patients who have lower education, heavy physical workloads, or limited family support, may strengthen the translation of knowledge and attitudes into sustained practice (26–28). In this context, spine centers may benefit from embedding rehabilitation coordinators or nurse-led teams who maintain proactive contact with patients during the vulnerable transition from hospital to home.

At the level of specific educational and behavioral interventions, the present KAP patterns suggest that programs should move beyond general messages about the importance of rehabilitation to focus on concrete competencies such as recognizing meaningful muscle fatigue, distinguishing normal postoperative discomfort from complications, and executing core stabilization and posture correction exercises accurately over time. KAP-based educational trials in lumbar disc herniation have shown that iterative, stage-specific education with practical demonstration, feedback, and periodic reassessment can enhance adherence and self-efficacy more than one-off didactic sessions (17, 18). Perioperative rehabilitation studies in joint disease likewise highlight the value of tailoring educational content and follow-up to individual readiness, preferred learning channels, and household responsibilities, with particular gains among patients who initially have lower health literacy (19, 22). In addition, research on determinants of physiotherapy compliance indicates that clear communication about rehabilitation goals, collaborative planning of exercise schedules, and discussion of likely barriers and coping strategies reduce attrition in the months following surgery (22–24). Aligning clinical practice with such evidence may require the development of structured, yet flexible, KAP-informed rehabilitation pathways that allocate more time to targeted instruction in identified weak knowledge domains, scheduled feedback on exercise performance, and problem-solving support for high-risk subgroups, rather than assuming that general encouragement will suffice to uphold long-term practice (19, 22).

This study has several limitations. First, convenience sampling from a single tertiary hospital may have introduced selection bias, restricted population representativeness, and limited the generalizability of the findings to broader postoperative populations. Second, the cross-sectional design precludes conclusions about temporal sequence or causality; CFA and SEM describe the fit of hypothesized associations but cannot establish causal pathways. Third, self-reported questionnaire data may be affected by recall bias, social-desirability bias, and other response biases despite the quality-control measures used to reduce inconsistent or patterned responses. Fourth, although two-round Delphi review, pilot reliability testing, and CFA supported the questionnaire’s content relevance, internal consistency, and construct validity, the instrument has not yet been externally validated in an independent or multicenter sample.

## Conclusion

In conclusion, postoperative patients demonstrated moderate knowledge and practice levels and positive attitudes. The observed associations highlight an attitude–practice gap but do not establish causal relationships. Routine care may benefit from stage-specific patient education, scheduled telephone or digital follow-up, periodic reassessment of exercise technique, and practical support from rehabilitation coordinators or nurse-led teams to help patients translate positive intentions into sustained rehabilitation behaviors.

## Data Availability

The original contributions presented in the study are included in the article/Supplementary material, further inquiries can be directed to the corresponding author.
