# Supplementary material for: Knowledge, attitudes, and practice regarding rehabilitation among postoperative patients with spinal degenerative diseases: a cross-sectional study
Source: Front Public Health. 2026 Sep 3;14:1885115. doi: 10.3389/fpubh.2026.1885115 (PMC13581909; doi:10.3389/fpubh.2026.1885115)
Supplement: Supplementary file 1 [file Table_1.docx]

**Supplementary Table S1. Fit indices for the corrected structural equation model**

| **Fit index** | **Reference criterion** | **Corrected SEM result** |
| --- | --- | --- |
| CMIN/DF | <3 acceptable | 2.564 |
| IFI | Approximately ≥0.90 | 0.918 |
| TLI | Approximately ≥0.90 | 0.907 |
| CFI | Approximately ≥0.90 | 0.917 |
