# Supplementary material for: Knowledge, attitudes, and practice regarding rehabilitation among postoperative patients with spinal degenerative diseases: a cross-sectional study
Source: Front Public Health. 2026 Sep 3;14:1885115. doi: 10.3389/fpubh.2026.1885115 (PMC13581909; doi:10.3389/fpubh.2026.1885115)
Supplement: Supplementary file 2 [file Table_2.docx]

**Supplementary Table S2. Fit indices for the confirmatory factor analysis**

| **Fit index** | **Reference criterion** | **CFA result** |
| --- | --- | --- |
| CMIN/DF | <3 acceptable | 2.607 |
| IFI | Approximately ≥0.90 | 0.915 |
| TLI | Approximately ≥0.90 | 0.905 |
| CFI | Approximately ≥0.90 | 0.915 |
| RMSEA | <0.08 acceptable | 0.065 |

*Note: KMO=0.947; Bartlett's test of sphericity, P<0.001; standardized factor loadings ranged from 0.405 to 0.863.*
